# Hygiene of the Eye

**Published:** 1891-03

**Authors:** 


### HYGIENE OF THE EYE.

The effort to accommodate the eye in looking at near objects requires the action of several muscles, which must continue to act so long as the sight remains fixed upon them. When the effort is long sustained, these muscles become weary; and when not given proper opportunity for rest, they become seriously diseased.

If the eyes become easily tired, and can be used but a short time without a blurring of vision or aching of the eye-balls, it is probable that there is some serious defect, and a competent oculist should be consulted.

Never try to read or do work requiring close application of the eye-sight, in a poor light. In reading, have the light come over the shoulder, the left, if convenient, and avoid using the eyes in a glaring light.

Avoid exposing the eyes to a sudden, bright light. When the eyes are opened after being closed for some hours, as on awaking from sleep, some little time elapses before they are fully accustomed to the light. On this account, it is not well to employ the eyes in reading immediately on waking in the morning.

Reading on the cars is injurious to the eyes on account of the shaking which continually changes the distance between the book and the eye, and thus taxes most severely the organs of accommodation. Reading when lying down is also injurious.

---

A SMALL piece of resin dipped in the water which is placed in a vessel on a stove, it is said, will add a peculiar property to the atmosphere of the room, which will give great relief to persons troubled with a cough. The heat of the water is sufficient to throw off the aroma of the resin, and gives the same relief that is afforded by combustion of the resin. It is preferable to combustion, because the evaporation is much more durable. The same resin may be used for weeks.

